# Cardiac hypertrophy at autopsy

**DOI:** 10.1007/s00428-021-03038-0

**Published:** 2021-03-19

**Authors:** Cristina Basso, Katarzyna Michaud, Giulia d’Amati, Jytte Banner, Joaquin Lucena, Kristopher Cunningham, Ornella Leone, Aryan Vink, Allard C. van der Wal, Mary N. Sheppard, on behalf of the Association for European Cardiovascular Pathology

**Affiliations:** 1 Cardiovascular Pathology Unit, Department of Cardiac, Thoracic and Vascular Sciences and Public Health, University of Padua, Padua, Italy; 2 University Center of Legal Medicine Lausanne - Geneva, Lausanne University Hospital and University of Lausanne, Lausanne, Switzerland; 3 Department of Radiological, Oncological and Pathological Sciences, Sapienza University of Rome, Rome, Italy; 4 Department of Forensic Medicine, University of Copenhagen, Copenhagen, Denmark; 5 Forensic Pathology Service, Institute of Legal Medicine and Forensic Sciences, Seville, Spain; 6 Department of Laboratory Medicine and Pathobiology, Ontario Forensic Pathology Service, University of Toronto, Toronto, Canada; 7 Cardiovascular and Cardiac Transplant Pathology Unit, Department of Pathology, Sant’Orsola-Malpighi University Hospital, Bologna, Italy; 8 University Medical Center Utrecht, Utrecht University, Utrecht, The Netherlands; 9 Amsterdam UMC, Academic Medical Center, Amsterdam, The Netherlands; 10 Department of Cardiovascular Pathology, Cardiology Clinical Academic Group, Molecular and Clinical Sciences Research Institute, St George’s Medical School, London, UK

**Keywords:** Autopsy, Cardiovascular diseases, Diagnostic criteria, Hypertrophy, Quality in pathology

## Abstract

Since cardiac hypertrophy may be considered a cause of death at autopsy, its assessment requires a uniform approach. Common terminology and methodology to measure the heart weight, size, and thickness as well as a systematic use of cut off values for normality by age, gender, and body weight and height are needed. For these reasons, recommendations have been written on behalf of the Association for European Cardiovascular Pathology. The diagnostic work up implies the search for pressure and volume overload conditions, compensatory hypertrophy, storage and infiltrative disorders, and cardiomyopathies. Although some gross morphologic features can point to a specific diagnosis, systematic histologic analysis, followed by possible immunostaining and transmission electron microscopy, is essential for a final diagnosis. If the autopsy is carried out in a general or forensic pathology service without expertise in cardiovascular pathology, the entire heart (or pictures) together with mapped histologic slides should be sent for a second opinion to a pathologist with such an expertise. Indication for postmortem genetic testing should be integrated into the multidisciplinary management of sudden cardiac death.

**Supplementary Information:**

The online version contains supplementary material available at 10.1007/s00428-021-03038-0.

## Introduction

The clinical and forensic pathologists are frequently faced with increased heart weight and heart size, with or without increased wall thickness or chamber dilatation at autopsy. The main challenge is to determine whether these features are physiological or pathological, and if they could represent the substrate for arrhythmogenesis or heart failure [1]. For example, in case of sudden death, when the only finding is increased cardiac mass, the pathologist should search for features which could suggest a possible genetic etiology and eventually advice the families for clinical and genetic counseling [2]. In clinical autopsies, especially those with complex multiorgan disease, the contribution of heart failure (either left or right sided, or both) to the death of a patient is frequently a matter of discussion.

Why is there a need for a document on the pathologic diagnosis of cardiac hypertrophy? This is a very important issue as cardiac hypertrophy could be considered as a cause of death. Assessment of the cardiac dimensions at autopsy requires a uniform approach, including terminology, a common methodology to measure the heart weight, size, and thickness as well as a systematic use of cut off values for normality. Interpretation of these findings needs a proper knowledge of the pathologic backgrounds of hypertrophy, including “new” diseases and adherence to the current insights obtained from clinical imaging (echocardiography, computed tomography—CT, cardiac magnetic resonance—CMR).

The diagnostic work-up in the setting of cardiac hypertrophy implies delineation of disease entities with hypertrophic hearts including pressure and volume overload conditions, compensatory hypertrophy, storage and infiltrative disorders, and cardiomyopathies. A number of gross morphologic features can indeed point to a specific diagnosis, such as concentric hypertrophy, biventricular hypertrophy, outflow tract obstruction, thickening of the interatrial septum, nodular thickening with calcification of the aortic valve, systemic and other organ involvement, and obesity.

However, in order to reach a final diagnosis, systematic histologic analysis is mandatory, integrated with special stains, immunostaining, and transmission electron microscopy (TEM) when appropriate.

This approach allows us to address the genetic form of hypertrophic cardiomyopathy (HCM) which is currently considered as “primary” cardiomyopathy, where the major distinctive feature is histological disarray of myocytes [3–5]. Finally, in some cases, the cause of hypertrophy remains unexplained after in-depth gross and histologic examination, and it should then be designated idiopathic left ventricular hypertrophy (LVH).

The goal of our paper is therefore to provide a uniform terminology and methodology to assess cardiac hypertrophy at gross and histologic examination of the heart, and also reference values available covering the range of “normal” cardiac measurements. This will allow to perform clinic-pathological correlation and to detect genetically determined forms.

## Terminology

The pathologist should be aware that cardiomegaly, cardiac hypertrophy, and cardiac dilatation are not interchangeable terms. The same applies for LVH and HCM. Patterns of myocardial hypertrophy, whether physiologic or pathologic, concentric or eccentric, symmetric or asymmetric, as well as regional “compensatory” hypertrophy should be clearly defined. Table 1 lists the terms currently used for gross and histologic features related to cardiac hypertrophy with proper definitions.

**Table 1:** Definition of terms

Terminology	Description/look for, helpful
Macroscopic	
Cardiomegaly	A big heart upon visual judgement of the size, transverse and longitudinal diameters can help
Cardiac hypertrophy	Increased heart weight compared to normal predicted values (see reference table and calculator)
Concentric hypertrophy	Increased heart weight without cavity dilatation, internal chamber diameter can help The wall thickness is usually increased
Eccentric hypertrophy	Increased heart weight with cavity dilatation, internal chamber diameter can help The wall thickness may be normal, increased, or decreased in eccentric hypertrophy.
Symmetric LV hypertrophy	Almost equal thickness of the entire LV and septum
Asymmetric LV hypertrophy	The thickness is not uniform but increased either in the free wall or in the septum. The most frequent variant is asymmetric septal hypertrophy with septal-to-posterior wall thickness ratio ≥ 1.3
Microscopic	
Myocyte hypertrophy	Increase in cardiac myocyte diameter > normal value. Nuclei are also increased in size irregular and hyperchromatic
Interstitial fibrosis	Increased deposition of connective tissue in the interstitium without myocytes loss. Special connective tissue stains can be helpful
Replacement fibrosis	Increased deposition of connective tissue following extensive myocytes loss. Special connective tissue stains can be helpful
Myocyte disarray	Loss of normal alignment of cardiac myocytes, often arranged at oblique and perpendicular angles to each other Can be either single myocyte or myocyte bundle disarray
Small vessel disease	Marked medial and or intimal thickening of intramural arterioles (100–500 micron) wall with luminal stenosis. Special connective tissue stains can be helpful

*Physiologic hypertrophy* is typically observed in people undergoing regular strenuous dynamic exercise (“athlete’s heart”) and during pregnancy. It is defined as a balanced type of eccentric hypertrophy.

*Pathologic hypertrophy* occurs due to volume and/or pressure overload, storage and infiltrative disease, systemic disorders, and cardiomyopathies. This leads mostly to concentric hypertrophy with wall and septal thickening and a loss of chamber cross-sectional area (mass/volume ratio increased). Over time, this state can deteriorate into a disproportionally increase in heart weight and dilated and eccentric forms of hypertrophy, leading to chamber enlargement with loss of free wall and septal thickness (mass/volume ratio decreased). In some diseases, particularly primary myocardial diseases (i.e., dilated cardiomyopathy-DCM) or valve incompetence (see for instance aortic incompetence), the cardiomegaly is characterized *in primis* by eccentric hypertrophy, with or without increased wall thickness, and without a preceding concentric remodeling phase.

## Methodology for macroscopic and histologic examination of the heart in relation to hypertrophy

### Heart weight

The heart should be weighted emptied of postmortem clots from both atria and ventricles, with aorta and pulmonary artery transected 1 cm above their origin, and without any attached tissue such as pericardium, mediastinal fat, or lung.

The heart weight is the first and important piece of information that indicates cardiac pathology [1, 2, 6]. The majority of hearts are weighed in the fresh state at autopsy. It should be noted that, although most values reported in the literature were based on measurements of formalin-fixed specimens, these values are applicable to fresh specimens because formalin fixation generally produces a slight change in heart weight, to return to the original fresh weight by three weeks [7, 8].

### Wall thickness

The heart is cut with a complete transverse (short-axis) section at the mid-ventricular level and then further parallel transverse slices of ventricles are performed at 1-cm intervals towards the apex [2]. These slices are assessed carefully looking for changes in the cut surface of the myocardium and the endocardium of the left ventricular (LV) and right ventricular (RV) cavities. We strongly recommend not to dissect the myocardium with slices parallel to the endo-epicardial surfaces because the opportunity for transmural, full thickness histological analysis gets lost. Then, the basal portion of the heart could be opened along the RV and LV outflow tract (LVOT) to assess the basal septum, the septal endocardium, and the anterior mitral valve leaflet with special attention to the geometric shape of the LVOT and presence of endocardial friction lesions.

The individual ventricular wall thickness should be measured at the mid-cavity level, between the cardiac base and apex, to the nearest 0.5 mm and must exclude trabeculae and epicardial fat [1, 2, 9, 10]. The measurements should be taken at the mid portion of the posterior wall of the LV and RV as well as mid-septum. The major challenge is to obtain an accurate measurement of the septum, given that pathologists frequently overestimate its thickness by incorporating trabeculae, such as the flattened septo-marginal trabeculation on the right side.

In the presence of asymmetric hypertrophy, more measurements will be taken to compare the septum and free wall segments at different sites.

Photographic documentation with a ruled reference scale should be made, before proceeding for histology.

When evaluating heart wall thicknesses at autopsy, postmortem phenomena such as rigor mortis or putrefaction have to be considered. It is well known that postmortem thickness could differ from echocardiographic values as measures are more in keeping with systolic than diastolic in vivo values [11]. Increased concentric thickness of the LV wall with small cavity and heart weight within normal limits could be related to rigor mortis [1]. With putrefaction, there will be discolouration and thinning of both RV and LV walls with artefactual chamber dilatation but in this case heart weight is usually normal or even diminished depending on the degree of putrefactive changes.

### Heart size

The transverse size (width) of the heart is best calculated as the distance from the obtuse to the acute margin at the posterior atrio-ventricular sulcus. The longitudinal size (length) is obtained as the distance between the crux cordis (i.e., the point at which the atrio-ventricular sulcus meets the posterior interventricular sulcus) and the apex of the heart on the posterior aspect [2, 12]. Measurements of ventricular chamber sizes have been proposed at the midventricular level at cross section but there is no clear consensus [13]. The assessment of atrial size at autopsy is even more challenging. Thus, besides the evaluation of chamber sizes, pathologic changes in the lung and liver, serous effusions, and peripheral edema can indicate acute or chronic heart failure at postmortem.

In each case, and particularly when dealing with cardiac hypertrophy, the aortic valve and aortic arch (isthmus) should be carefully investigated to exclude causes of pressure overload.

**Fig. 1:**
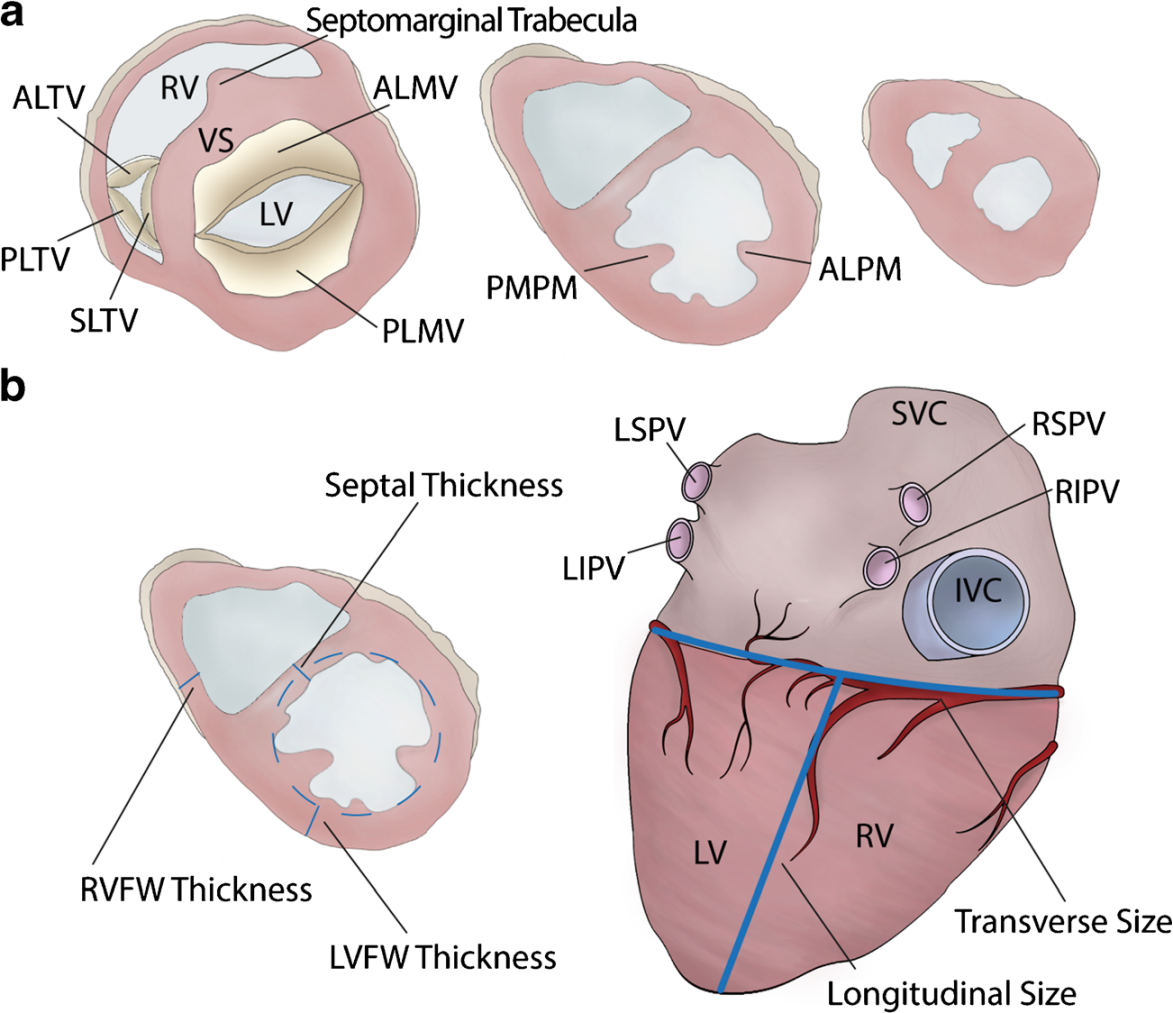
**a** Diagrams illustrating the three cross sections of the hearts: the first mid-ventricular one and at least two additional parallel sections towards the apex. **b** *On the left*: left ventricular (posterior), septal (middle), and right ventricular (posterior) thickness measurements, by excluding trabeculae and papillary muscles. *On the right*: transverse size and longitudinal size measurements on the posterior aspect of the heart. ALMV, anterior leaflet mitral valve; ALPM, antero-lateral papillary muscle; IVC, inferior vena cava; LV, left ventricle; LVFW, left ventricular free wall; LSPV, left superior pulmonary vein; LIPV, left inferior pulmonary vein; PLMV, posterior leaflet mitral valve; PSPM, postero-septal papillary muscle; RIPV, right inferior pulmonary vein; RSPV, right superior pulmonary vein; RV, right ventricle; RVFW, right ventricular free wall; SVC, superior vena cava; VS, ventricular septum

Diagrams to illustrate how to measure the wall thicknesses and cardiac size are illustrated in Fig. 1.

### Size of the heart in postmortem imaging

A first idea of heart size can be obtained radiologically by measuring the cardiothoracic ratio (CTR). The postmortem CTR is influenced by body mass index (BMI) and the heart dilatation that could be related to perimortem phenomena [14] and cannot be used alone to discriminate between normal and increased heart mass. A formula of adjusted CTR-based score could help to predict “cardiomegaly” at postmortem computed tomography (PMCT) [15]. Several studies showed that postmortem radiological measurements of wall thicknesses including PMCT and postmortem magnetic resonance (PMMR) differ from corresponding autopsy measurements [16–20]. It was shown that LV wall thickness was significantly higher on PMCT compared to PMMR and that LV anterior wall thickness was significantly higher on PMMR than at autopsy [17]. PMCT was suggested as a helpful tool for the assessment of LV mass, which is important in clinical practice [21]. Further work is needed to correlate postmortem imaging with autopsy values.

### Histology and additional tools

Standard histologic examination of the myocardium requires mapped labeled blocks (variable in number depending on the size of the heart) from a representative transverse slice of the ventricles to include the LV wall (usually anterior, lateral, and posterior), the ventricular septum (usually anterior, middle, and posterior), and the RV free wall (usually anterior, lateral, and posterior), as well as RV outflow tract [2]. LV myocardial sections should include the papillary muscles. Additionally, one block from any area with significant macroscopic abnormalities should be taken. Hematoxylin and eosin and a connective tissue stain (elastic van Gieson, trichrome, or Sirius red) are routinely performed. Other special stains and immunohistochemistry should be performed as required particularly if there is a suspicion of storage or infiltrative disorders. The appropriate immunohistochemistry stains to diagnose specific forms of storage and infiltrative disorders are reported elsewhere [22]. TEM can be of help in the latter conditions as well as in mitochondrial disorders. Ideally, if one is suspecting an infiltrative, storage, or mitochondrial disorder, samples of myocardium should be placed in glutaraldehyde at the time of autopsy as working later from paraffin-embedded tissue may only have limited success. However, this is not feasible in routine practice.

## Definition of normal heart in relation to hypertrophy

### Gross examination

Transverse and longitudinal dimensions of the heart are useful parameters to judge cardiac size; however, normal values according to body size are mostly missing. According to *Gray’s Anatomy*, the heart length and width are 12 cm and 8.5 cm [12]. More recently, length and width have been investigated in different age groups in the Iranian population, with mean values of 11.41 ± 2.15 cm and 8.21 ± 4.38 cm, respectively [23].

However, the heart weight is the most important parameter in the determination of cardiac hypertrophy. The obtained heart weight value should be compared against tables of normal weights by age, gender, and body weight and height [6, 9, 10]. The weighing of the body and measuring of the height are essential in all autopsies. Other anthropometric data, such as abdominal circumference that are related to cardiovascular risk factors, can be taken but the clear relationship with the heart weight has not been established yet. There are many studies proposing normal values for heart weights, with a large variability of methodology and obtained values (Supplemental Table 1) [9, 24–38]. It is stressed in the literature that reference tables should be established for local population and periodically updated [6, 25]. The most frequently cited reference values for the heart weight are from Zeek et al. and Kitzman et al. [9, 35], and are based on the data published respectively in 1942 and 1988 concerning the North American population.

More recently, it has been shown in the Swiss population that the heart weight increases along with the increase of the body weight, body height, BMI, and body surface area (BSA). The mean heart weight is greater in men than in women at a similar body weight. The reference tables for predicted heart weights were presented as a user-friendly internet application (http://calc.chuv.ch/Heartweight) enabling the comparison of heart weights observed at autopsy with the reference values [6].

If it is not possible to establish the predicted values, 500 g in adults male and 400 gr in adult female may be considered a reasonable cut-off [39].

**Fig. 2:**
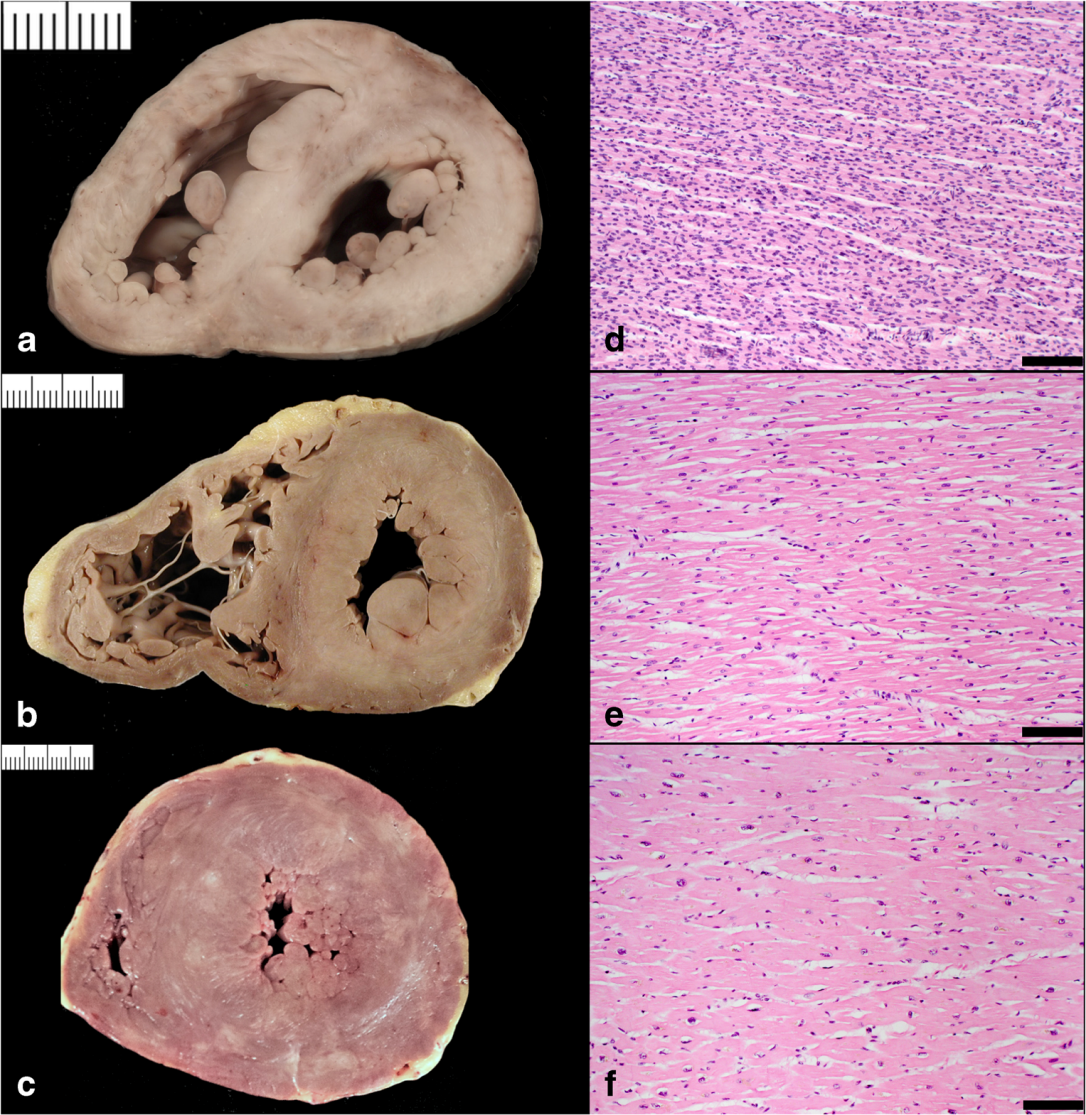
Normal heart vs. cardiac hypertrophy. **a** Cross section of a normal heart in a perinatal death: the right ventricular free wall thickness is 3.5 mm, the left ventricular is 5 mm, and the septum 5.5 mm. **b** Cross section of a normal adult heart: the right ventricular free wall thickness is 2 mm, the left ventricular is 12 mm, and the septum 13 mm. **c** Cross section of hypertrophic heart in an adult: the right ventricular free wall thickness is 7 mm, the left ventricular is 21 mm, and the septum 22 mm. **d** Histology of **a** showing hypercellularity which is normal for a perinatal myocardium (high number of cardiac myocyte/myocardial area) (bar = 100 micron). **e** Histology of **b** with diameter of cardiac myocytes within normal values (mean diameter 12 micron) (bar = 100 micron). **f** Histology of **c** with cardiac myocyte hypertrophy (mean diameter 20 micron) (bar = 100 micron)

Ventricular wall thicknesses for adults from 20 to 99 years of age were published by Kitzman et al. [9]. The mean thickness of the RV free wall was 3.8 mm (SD; 0.9 mm) and the LV free wall was 12.3 mm (SD 1.6 mm). The mean ventricular septal thickness was 13.6 mm (SD 2.0 mm) (Fig. 2). The ventricular wall thicknesses were similar for women and men and thickness of the RV and LV free walls, indexed by BSA, remains relatively constant through adult life. An appreciable increase in indexed ventricular septal thickness was observed through to the 10th decade of life. Summing up, we can consider abnormal values those exceeding the reference range, i.e., a LV wall thickness >14 mm, a RV wall thickness >5 mm, and a ventricular septal thickness >15 mm [1, 40].

In children, predicted normal ventricular wall thicknesses for females and males under the age of 20 years were published by Scholz et al. in 1988 [10]. In this age group, the mean (and upper 95%) values are presented in the Supplemental Table 2.

### Histology

The myocardium is composed of cross-striated muscle cells (cardiac myocytes) enveloped in a loosely arranged extracellular matrix (interstitium). The cardiac myocytes account for about two-thirds of the myocardium by cell volume but only for one-third by cell number. A healthy adult cardiomyocyte is cylindrical in shape and measures approximately 100 μm in length (the myocyte length is impossible to measure on routine sections) and up to 15 μm in diameter; the best way to measure the diameter is on cross section when the nucleus is located centrally in the muscle cell [41, 42] (Fig. 2). However, these values should be further evaluated in future, by applying digital microscopy systematically on cardiomyocyte sizes in relation to heart weights and age at autopsy. The interstitium contains microvessels and also some extravascular cells such as fibroblasts, macrophages, lymphocytes, and mast cells.

## Patterns of cardiac hypertrophy

**Fig. 3:**
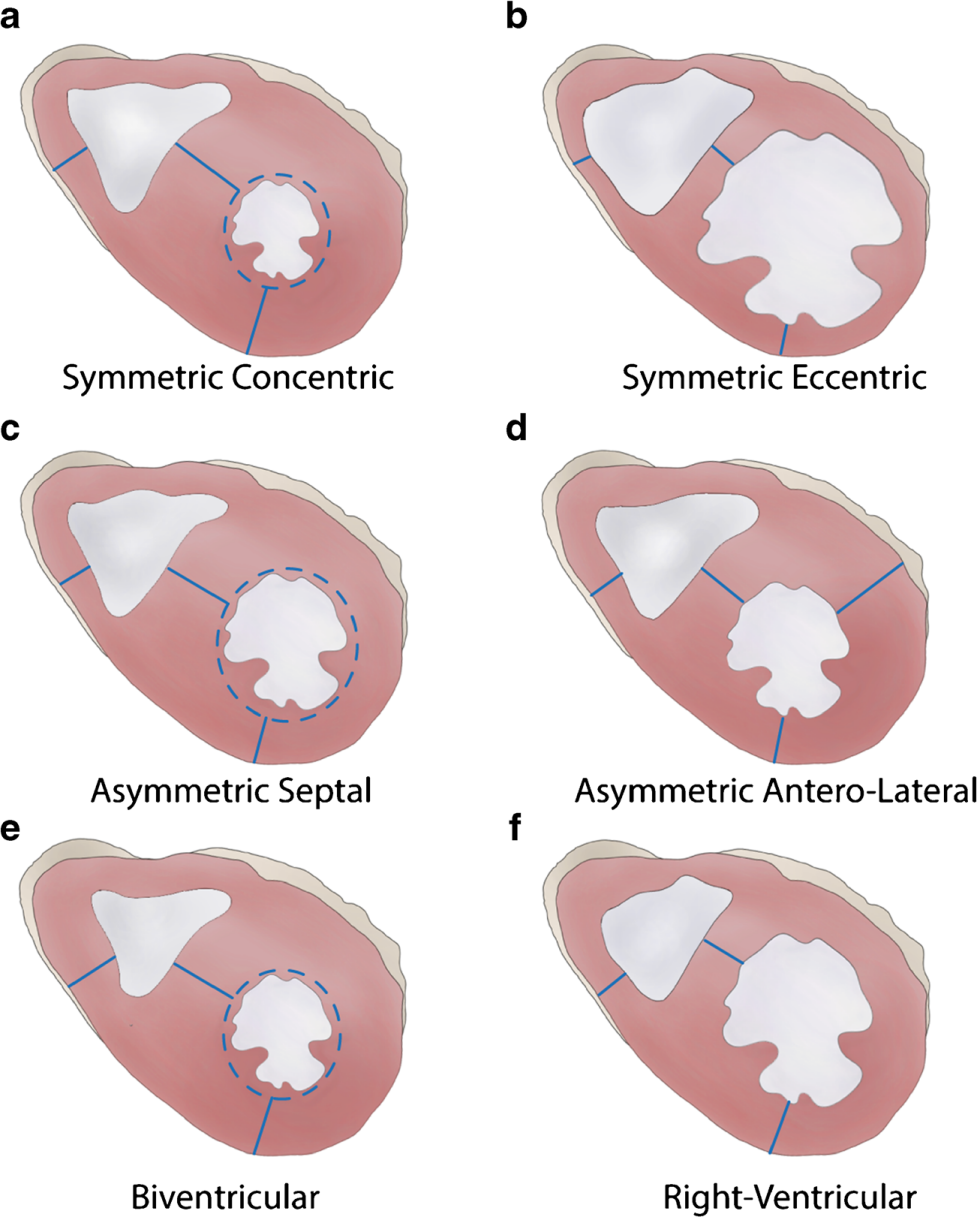
Diagrams illustrating various patterns of cardiac hypertrophy. **a** Symmetric, concentric. **b** Symmetric, eccentric. **c** Asymmetric septal. **d** Asymmetric antero-lateral. **e** Biventricular. **f** Right ventricular

**Fig. 4:**
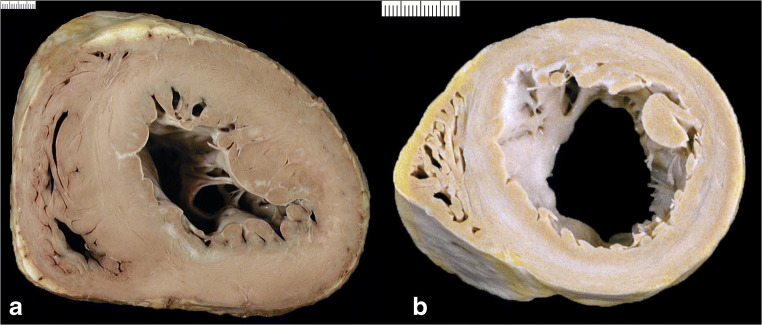
Cardiac hypertrophy. **a** Concentric type (with increased LV wall thickness). **b** Eccentric type (with increased LV cavity)

**Fig. 5:**
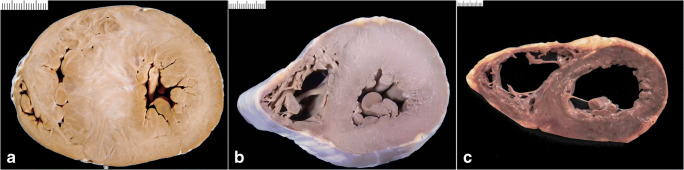
“Primary” cardiomyopathies. **a** Asymmetric septal hypertrophic cardiomyopathy with septal/LV free wall thickness ratio >1.3. **b** Asymmetric antero-lateral hypertrophic cardiomyopathy. **c** Dilated cardiomyopathy with biventricular dilatation

**Fig. 6:**
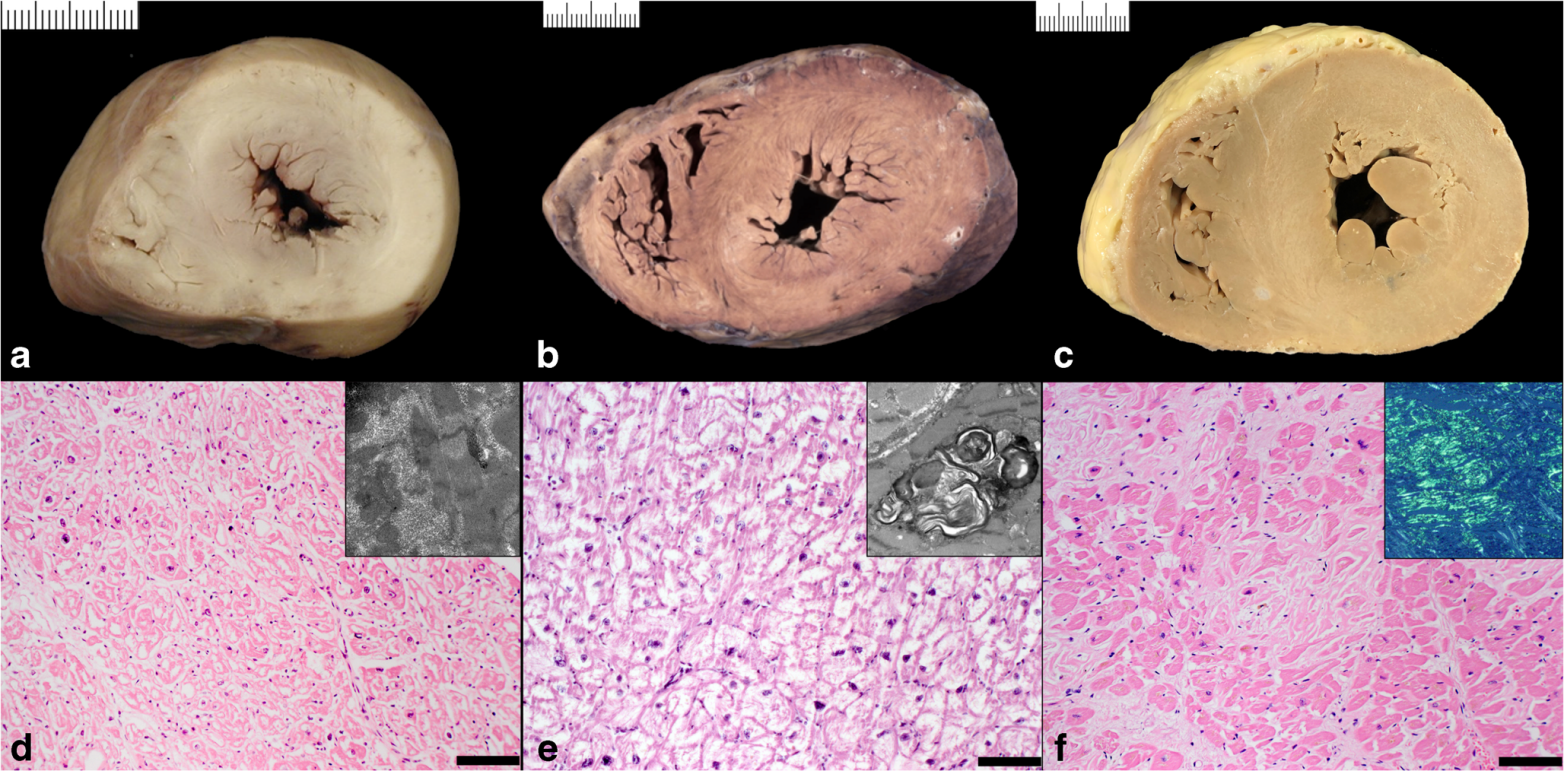
“Secondary” cardiomyopathies. **a** Concentric biventricular hypertrophy in storage disease/glycogenosis. **d** Histology of **a** showing diffuse cardiac myocytes vacuolization (hematoxylin eosin stain bar = 100 micron; insert TEM with intracellular glycogen deposits). **b** Concentric biventricular hypertrophy in storage disease/Fabry disease. **e** Histology of **b** showing extensive vacuolization of the cardiac myocytes (hematoxylin eosin stain bar = 100 micron; insert TEM with concentric lamellar bodies). **e** Concentric biventricular hypertrophy in infiltrative disease/amyloidosis. **f** Histology of **c** with amorphous material deposition in the interstitial space (hematoxylin eosin stain bar = 100 micron; insert shows the amorphous Congo red–positive substance at polarized light)

**Fig. 7:**
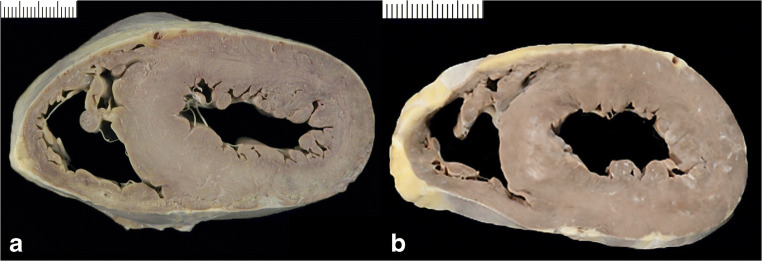
Cardiac hypertrophy due to pressure overload. **a** Hypertensive heart disease. **b** Aortic valve stenosis due to dystrophic calcification

**Fig. 8:**
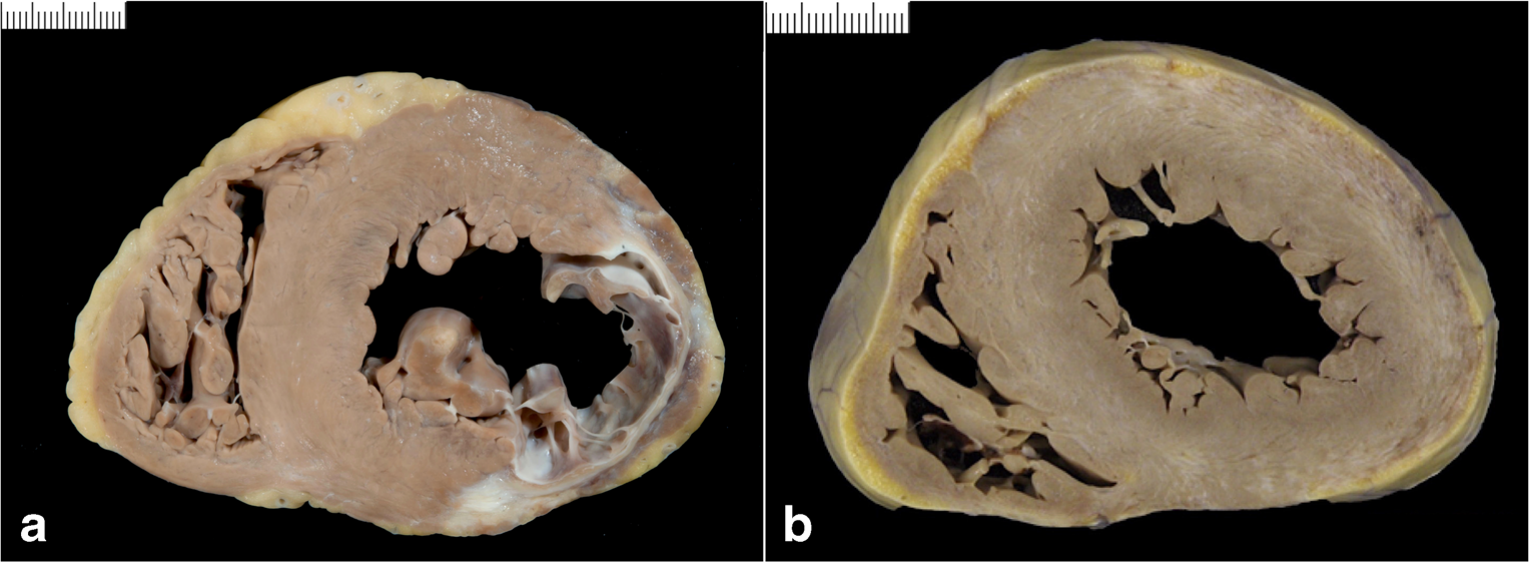
Compensatory hypertrophy. **a** Chronic ischemic heart disease with previous lateral myocardial infarction. **b** Subacute-chronic myocarditis with mild left ventricular hypertrophy

**Fig. 9:**
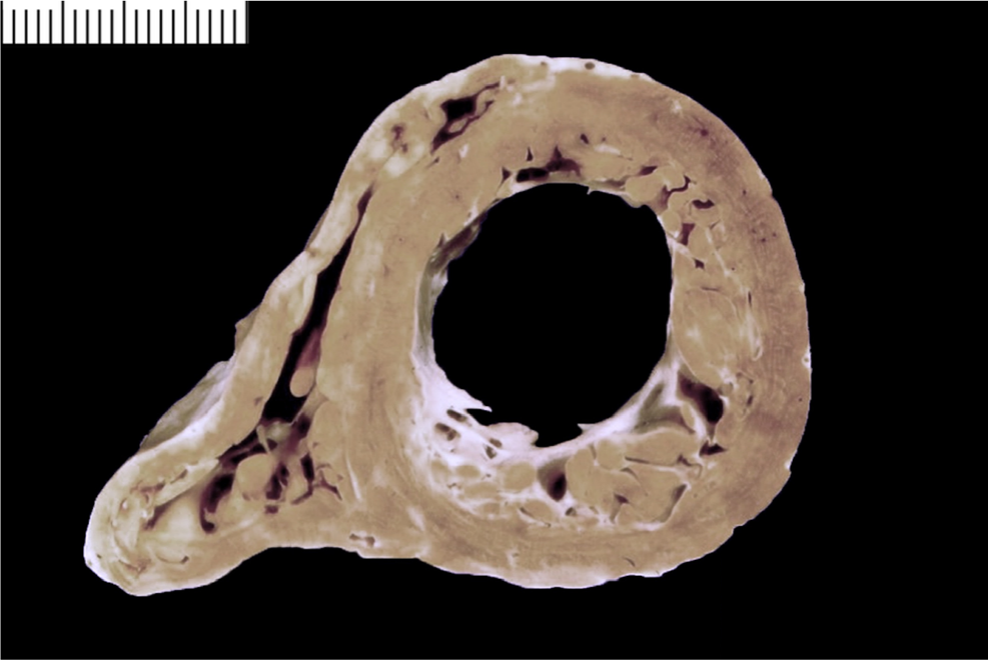
Left ventricular noncompaction cardiomyopathy/dilated. Note the left ventricular cavity enlargement, thinning of the compact layer with prominent trabeculae of the left ventricular free wall. Note the endocardial fibroelastosis following the outline of the trabeculae

**Fig. 10:**
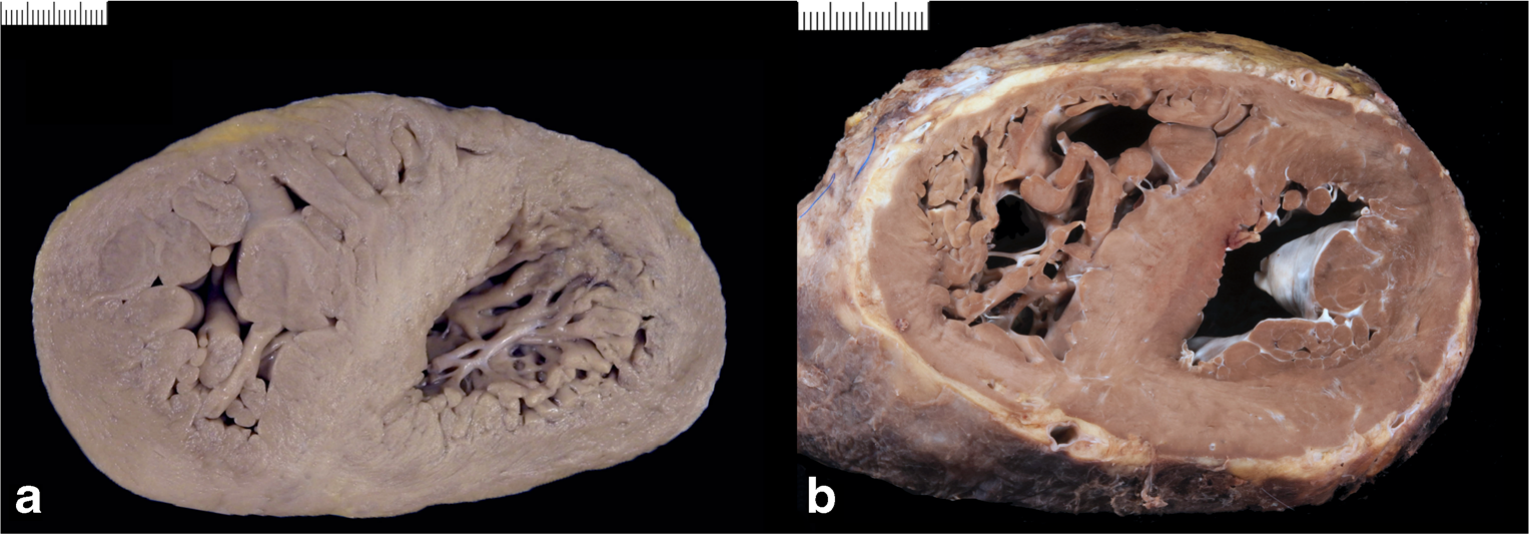
Right ventricular hypertrophy. **a** Primary pulmonary hypertension. **b** Congenital heart disease (surgically repaired atrio-ventricular septal defect)

Diagrams of **macroscopic patterns** of cardiac hypertrophy as defined in Table 1 are exemplified in Fig. 3. Gross pictures of hearts with various patterns of cardiac hypertrophy are illustrated in Figs. 4, 5, 6, 7, 8, 9, and 10. Macroscopic features that should be considered in the differential diagnosis of the various conditions leading to LVH are reported in Table 2.

**Table 2:** Macroscopic features in different conditions accounting for left ventricular hypertrophy

	Hypertrophic cardiomyopathy	Hypertensive heart disease	Aortic stenosis, coarctation	Amyloidosis^#^	Storage disease^#^	Drug induced	Ischemic heart disease	Athlete heart
Atrial septum thickening	-	-	-	+	+	-	-	-
RV hypertrophy	+	-	-	+	+	-	-	-
Asymmetric LV hypertrophy	+	+	-	+	+	-	+	-
Symmetric LV hypertrophy	+	+	+	+	+	+	-	+
LVOT bulging	+	+	+	-	-	-	-	-
LVOT endocardial plaque	+	-	-	-	-	-	-	-
Scarring	+	+	+	+	+	+	+	-
Papillary muscle abnormalities	+	-	-	-	-	-	-	-
AV valve thickening	-	-	-	+	+	+	-	-
Endocardial wrinkling	-	-	-	+	-	-	-	-

Malformation syndromes, neuromuscular disorders, and mitochondrial diseases are excluded (clinical history, external examination, and other organs involvement usually help). ^#^Other organ/tissue involvement

For an overview of causes of cardiac hypertrophy, see Elliott P. et al. [5]

+ stands for can be seen

RV hypertrophy is usually seen in the setting of left-sided heart diseases, pulmonary diseases, and congenital heart diseases. It may also occur in HCM, Anderson-Fabry, and Noonan syndrome.

### Microscopic patterns

**Fig. 11:**
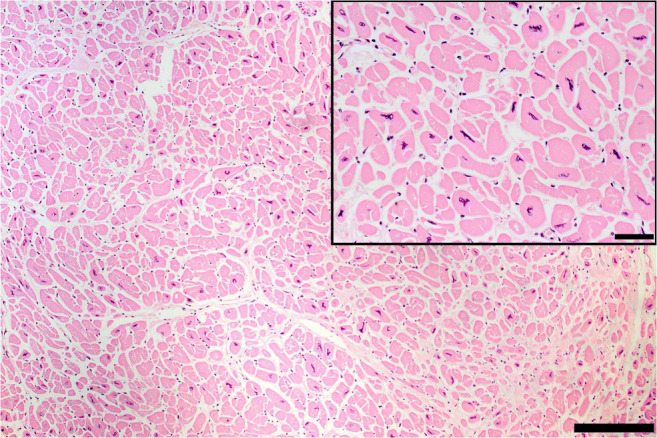
Histology of cardiac myocyte hypertrophy. Note the increased cardiac myocyte diameter and the bizarre, irregular, and hyperchromatic nuclei (hematoxylin eosin stain bar = 50 micron). A close-up (bar = 100 micron) is seen in the insert

The term myocyte hypertrophy refers to an increase in cardiac myocyte diameter >15 μm, measured in cross section at the nuclear level. Other histologic characteristics of cardiac myocyte hypertrophy are the presence of nuclei which are also increased in size, irregular, and hyperchromatic due to increased DNA ploidy resulting from DNA replication in the absence of cell division (Fig. 11).

Table 1 lists the histologic features with proper definitions related to the wide spectrum of acquired and genetic forms of cardiac hypertrophy, as mentioned before.

With the exception of storage, infiltrative, and cardiomyopathies, in all conditions of adaptive hypertrophy (pressure/volume overload or compensatory), the only constant feature is cardiomyocyte hypertrophy.

## Cardiac hypertrophy according to pathophysiology

### Primary cardiac hypertrophy (HCM and DCM)

**Fig. 12:**
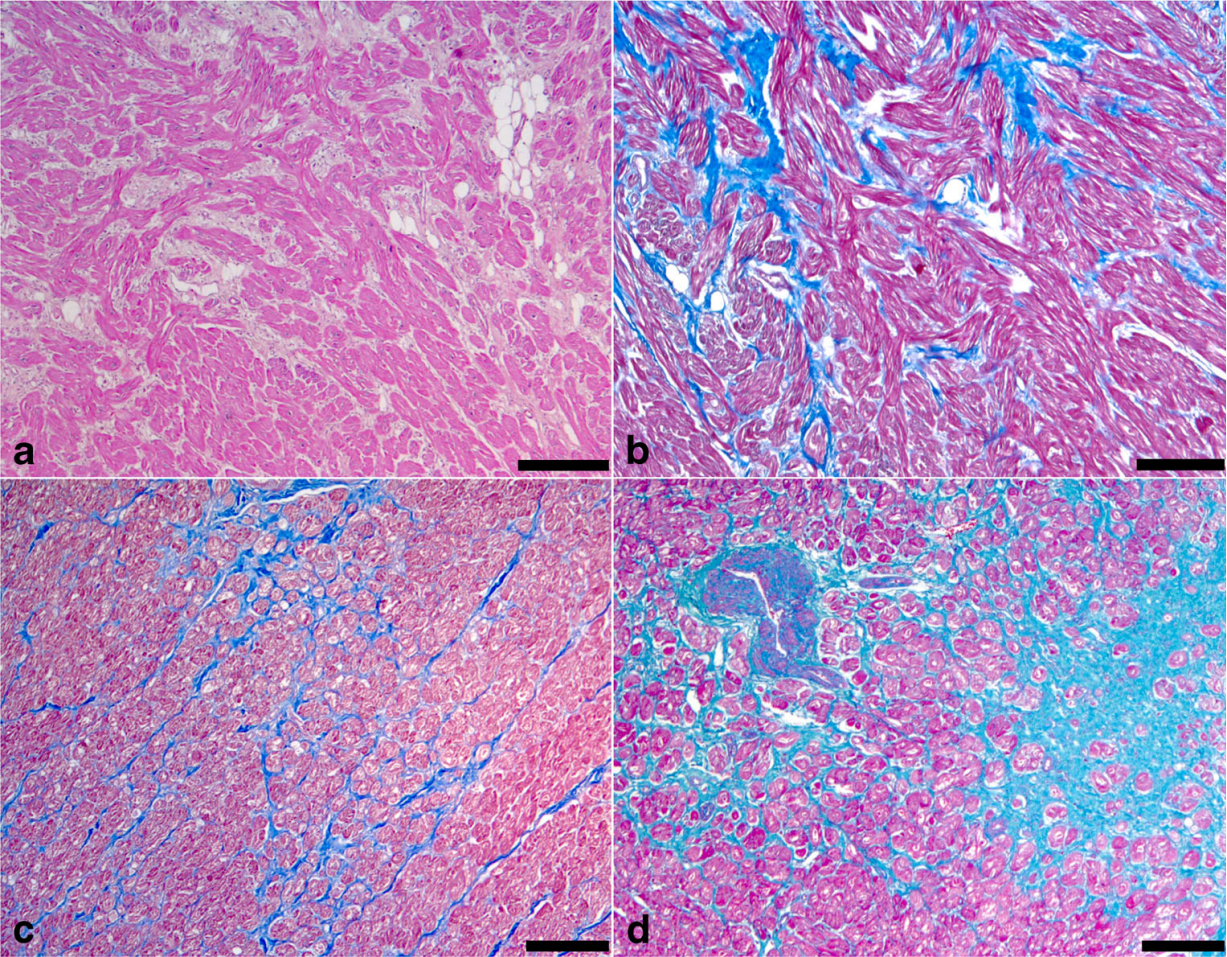
Histology of hypertrophic cardiomyopathy. **a** Myocyte bundle disarray (hematoxylin eosin stain). **b** Myocyte bundle disarray (trichrome stain). **c** Interstitial fibrosis (trichrome stain). **d** Replacement-type fibrosis with small vessel disease (trichrome stain). All bar = 100 micron

**HCM** is the most common genetically determined primary heart muscle disease, affecting 0.2–0.5% of the general population. The pattern of inheritance is autosomal dominant, mostly due to mutations in genes encoding for sarcomeric proteins (most often β-myosin heavy chain and myosin-binding protein C). HCM is characterized by extreme phenotypic heterogeneity with either symmetric or asymmetric pattern of LVH. In the latter, the interventricular septum is markedly thickened relative to the LV free wall, although the free wall can also exhibit asymmetric hypertrophy (Fig. 5). Subaortic bulging leading to LVOT obstruction, with or without septal endocardial plaques (“friction lesion”), can be observed. A significant variability in the regional wall thickness is reported [43, 44]. Less common variants of HCM include those with regional wall thickenings involving the mid-septal wall, the apex, the anterior-lateral, and posterior-basal free wall. All LV segments from base to apex have to be examined since LVH can be confined only to one or two segments. The disease can occasionally progress to heart failure (end-stage HCM) due to extensive scarring with a markedly dilated ventricular cavity [45]. A grossly visible whorled cut surface, reflecting the histologic disarrangement of cardiac myocytes, is sometimes appreciable when examining the heart either fresh or after fixation. Scarring can be more evident after fixation (supplemental figure). Myocyte hypertrophy, architectural disorganization (so-called disarray), and interstitial fibrosis are histopathologic features of HCM (Fig. 12). However, myocyte disarray is not pathognomonic of HCM, and can also be observed in other heart diseases, including congenital heart diseases, as well as in normal hearts, where it is physiological at the ventricular free wall–septal junctions or in the trabeculae [46, 47]. The existence of HCM with normal heart weight and thickness (i.e., HCM without hypertrophy) but with widespread myocyte disarray at histology is recognized [48]. Myocardial bridge (i.e., a deep intramyocardial course of the left anterior descending coronary artery) is much more frequent in HCM than in the normal heart [49]. Intramural small vessel disease, due to cellular or fibrous intimal thickening and medial hypertrophy or fibrosis, is another common histopathologic finding in HCM. Ischemic myocardial injury, with either myocyte necrosis or chronic fibrous scars, is often present [50]. Although in most cases HCM can be discriminated from secondary hypertrophy, e.g., due to hypertension, this is not always the case. Especially in hypertrophic hearts with symmetric hypertrophy and without significant myocyte disarray, the discrimination can be difficult by histopathology alone. In all these cases where doubt remains after the heart examination, it is very important to advice additional molecular autopsy in the context of screening of first-degree family members via a cardiogenetic center to further evaluate the diagnosis of familial HCM.

**DCM** encompasses etiologically heterogeneous myocardial disorders that are defined by increased heart size, LV or bi-ventricular dilation with decreased ejection fraction, in the absence of hypertension, valve disease, and coronary artery disease. DCM prevalence reaches 1/250–500 in clinical practice. A positive familial history can be detected in up to 30–50% of cases, and a genetic determinant can be identified in up to 40% of cases. Sarcomeric, mitochondrial, and neuromuscular disorders are frequent etiologies in the presence of a familial history, where additional acquired conditions like exposure to toxics, diabetes, arrhythmia, myocarditis, and pregnancy can also account for DCM.

Macroscopically, increased heart weight and size with preserved or decreased LV wall thickness are typical of DCM (Fig. 5). Endocardial fibrous thickening can be observed in long standing cases due to thrombus organization. Mural thrombi can be found. Endocardial fibroelastosis can be present both in adults and children.

Myocyte hypertrophy can be seen at histology, together with degenerative changes/cytoplasmic rarefaction/vacuolization and irregular/abnormal nuclei with perinuclear halo [51–53]. Interstitial fibrosis with or without replacement type fibrosis is described. There is no direct match between the pathologic findings and the etiopathogenesis, so that for instance alcoholic cardiomyopathy and DCM of other causes are not distinguishable.

### Cardiac hypertrophy due to storage or infiltrative diseases (Fig. 6)

There are specific heart muscle diseases that may account for either concentric or eccentric cardiac hypertrophy in the setting of systemic disorders. They include disorders that may be localized to the myocardial interstitium (amyloidosis) or within myocardial cells (Iron Heart Disease-Hemochromatosis; storage disease such as Anderson Fabry’s disease with deposits of ceramide trihexoside, Pompe’s disease—type II glycogen storage disease, Gaucher’s disease with glucosyl ceramide lipidosis). The detailed presentation of these entities is out of the scope of this document. For an overview of causes of cardiac hypertrophy, we suggest to check the pertinent literature [5]. Special histological stains and, in selected cases, TEM are crucial for the final diagnosis.

### Cardiac hypertrophy due to pressure overload (concentric hypertrophy) (Fig. 7)

- Hypertensive heart disease. At autopsy, by far the most common form of LVH relates to systemic hypertension. This results from longstanding pressure overload due to increased peripheral resistance. The hypertensive heart frequently shows coexistence of atherosclerotic coronary artery disease and enlarged left atrium. With time, significant hypertension with gradual stiffening of the LV wall results in eccentric type of hypertrophy with concomitant dilatation of the cavity and eventually a pattern difficult to discriminate from DCM [54].
- Valve diseases. Similar to hypertension, any obstructive disease of the LVOT and aorta causes LV pressure overload. These include aortic valve stenosis (congenital-unicuspid, bicuspid, and tricuspid- or acquired-senile dystrophic calcification or rheumatic), sub- and supravalvular stenosis, and aortic isthmic coarctation. Also similar to hypertension, LVOT obstructions may end up in a volume overload type of LVH with chamber dilatation.

### Hypertrophy due to volume overload (eccentric hypertrophy)

Physiologically it occurs in endurance athletes (running, swimming) and during pregnancy, due to increased preload.

Pathologically, all diseases leading to aortic valve regurgitation and/or mitral valve regurgitation, which in both cases can be either acquired or congenital, can lead to volume overload with severe chamber dilatation and decreased mass/volume ratio. Atrial dilatation can be helpful in the assessment of valve incompetence.

### Other conditions of compensatory hypertrophy (Fig. 8)

Heart hypertrophy is a common finding in dilated hearts due to significant ischemic scarring (postmyocardial infarction chronic ischemic heart disease) and inflammatory or infiltrative cardiomyopathies. In these instances, hypertrophy compensates for the loss of vital myocardium. In particular, in the setting of ischemic heart disease, regional wall changes are a common feature, when segments of myocardium adjacent to the scarred area undergo a “compensatory” hypertrophy.

Papillary muscles and infero-basal LV wall hypertrophy and fibrosis, due to localized hypercontractility triggered by the prolapsing leaflet, have been reported in mitral valve prolapse [55, 56].

Compensatory hypertrophy of the remaining myocardium has also been described in arrhythmogenic cardiomyopathy. Hypertrophy of the trabeculae is a well-known epiphenomenon accompanying subepicardial, midmural, or transmural fibro-fatty replacement of the ventricular myocardium [57–59].

### Cardiac hypertrophy with a complex/mixed pathophysiology

#### Diabetes

Diabetic heart disease is a distinct pathology independent of co-morbidities such as coronary artery disease and hypertension. Cardiac remodeling is influenced by hyperglycemia, dyslipidemia, and hyperinsulinemia which promote β-adrenergic signaling leading to hypertrophy and eventual cardiac failure. Additionally, volume-loading from obesity can contribute to the cardiac hypertrophy [60].

#### Obesity

Obesity-associated LVH can be explained by increased blood cardiac output due to longstanding excess of body weight, and probably high metabolic activity of the large load of fat tissue. It occurs especially in patients presenting morbid obesity, which may lead to the so-called obesity cardiomyopathy with congestive heart failure. Additionally, hypertension and obstructive sleep apnea contribute to the cardiac hypertrophy in the morbid obese. Normal weight, concentric and eccentric types of LVH, and biventricular hypertrophy and excess of epicardial fat and fatty infiltration of RV have been reported [61].

#### Congenital heart disease

The spectrum of congenital malformations of the heart and great vessels is highly diverse, with either shunts between pulmonary and systemic circulation, abnormal insertions of vasculature or obstructive/stenosing lesions as their main features. They can occur as single lesions or in combinations, but invariably lead to significant aberrant hemodynamic flow patterns in the heart. Adaptive changes start early after birth, and involve one, two, three, or four of the cardiac chambers depending on the type of underlying malformation (Fig. 10). At present, most patients with congenital heart disease undergo surgical correction or palliation with survival until adult age. However, nearly all these patients show at least to some extent a remaining morbidity, which relates to the initial lesion or the surgical repair. Detailed patterns of hypertrophic remodeling for each type of malformation have been recently reviewed [62].

#### Drugs

Cardiac structural abnormalities in drug abusers are observed essentially in chronic cocaine addicts, and include LVH with increased heart weight, small vessel disease, and atherosclerotic coronary artery disease [63]. Different hypotheses have been postulated to explain LVH in cocaine abusers, such as the transient elevation of systolic blood pressure after cocaine use or the direct stimulation of myocardial α-adrenergic receptors [64]. Amphetamine and its derivatives as ecstasy cause an identical type of cardiotoxicity as cocaine [65]. Androgenic anabolic steroids are known to cause cardiac hypertrophy with increased mass, fibrosis, and substantial impairment of LV systolic and diastolic function [66].

## Gray zones

### Athlete’s heart

Intense and chronic athletic training is associated with LV remodeling, including an increase in wall thickness, cavity size, and mass. The extent of morphological cardiac changes depends on a variety of factors such as body size, gender, discipline of sport, ethnicity, and, likely, genetic factors. The type of training (endurance vs. strength training) is of special importance. Endurance exercise predominantly produces volume load on the LV and strength exercise causes largely a pressure load. The adaptations to intensive endurance or strength training can cause the so-called athlete’s heart [67]. These changes are considered generally as physiological and reversible in most cases. However, training-induced modifications may mimic the remodeling of pathological hypertrophy and the differential diagnosis between the athlete’s heart and some pathological conditions as HCM may be challenging [68].

### Cardiac hypertrophy in people of African descent

LVH is more common in this ethnic group, and is often associated with a greater severity of arterial hypertension [69]. Arterial hypertension is due to a combination of genetic, endocrine, and hemodynamic factors [70].

### Basal-septal hypertrophy in the elderly

In a subset of elderly normal subjects, with normal wall thickness elsewhere, basal septum hypertrophy is an age-related anatomic variant [71].

### Left ventricular noncompaction (or hypertrabeculation) (Fig. 9)

It is characterized by one or more LV segments, and sometimes biventricular, with excess trabeculations and deep intertrabecular recesses making up 2/3 of the wall with thinning of the compact layer. Papillary muscles need to be excluded when measuring the noncompacted layer. In children it can be seen in association with congenital heart disease and pediatric cardiomyopathies like Barth syndrome. In adults it shares phenotypic overlap with DCM and HCM and it is still debated whether it should be considered as a separate entity [72, 73]. The genetic basis is heterogeneous, often coinciding with that of cardiomyopathies and/or congenital heart disease [74].

### Idiopathic LVH

It is defined as LVH, usually concentric, at gross examination with hypertrophic cardiac myocytes in the absence of disarray at histology after extensive sampling. There is no history of hypertension or other explanations for the LVH [75, 76].

## Right ventricular hypertrophy (Fig. 10)

The mechanisms that lead to the distinct forms of LVH will obviously also apply to the RV. Moreover, both ventricles cannot be seen as separate entities. Hypertrophy of either the RV or LV alters the shape of the ventricular septum which may have significant effects on the ventricular geometry.

Primarily RV *pressure overload* may be due to pulmonary valve stenosis, primary pulmonary hypertension, chronic pulmonary disease, or left-sided heart disease with left heart failure. Chronic pressure overload leads to myocardial hypertrophy, which is essentially of concentric type. However, on the longer term also, volume adaption ensues, which means that at autopsy many cases show in fact an eccentric type of RV hypertrophy with dilated ventricle. In such cases, the interventricular septum has an almost straight configuration, which affects the LV geometry.

Pseudo-hypertrophy of the RV when epicardial fat merges into underlying myocardium can occur in the setting of obesity and arrhythmogenic cardiomyopathy, in the latter associated with fibrosis.

## Acute inflammation or ischemia

Increased ventricular and septal wall thickness due to massive interstitial edema with preserved cardiac myocytes size (“pseudo-hypertrophy”) can be found in the setting of acute myocarditis or ischemic reperfusion injury such as after prolonged cardiac arrest with resuscitative maneuvers. Histology and clinical history can be of help for differential diagnosis [77, 78].

## Referral for second opinion in cardiovascular pathology

If the autopsy is carried out in a general or forensic pathology service without expertise in cardiovascular pathology, the entire heart should be retained and sent to a specialized center with that expertise (reference network) to support routine workup [2, 79]. The referring pathologist/forensic performing the autopsy should examine the heart in a conservative way so that all the main structures are still appreciable in case of referral. If the heart cannot be retained then cardiac samples taken as indicated above should be processed to slides and sent for an expert opinion. It is essential that extensive photographic documentation with a ruled reference scale is made, indicating where individual blocks are taken. At least one transverse section of the heart including the LV and RV should be retained/sampled circumferentially for further examination.

## Postmortem genetic testing

Indication for postmortem genetic testing should be integrated into the multidisciplinary management of sudden cardiac death [80]. The finding of a hypertrophied heart at postmortem opens the doors to a potentially inherited cardiovascular disease and first-degree family members should be referred for clinical/genetic counseling [2]. The collection and storage of frozen of blood or tissue samples should be always guaranteed in the course of an autopsy, together with detailed cardiac pathology phenotype, for possible genetic testing in case of a suspected inherited disease.

## Supplementary Information

ESM 1 Hypertrophic cardiomyopathy, gross examination of the heart in the autopsy room and after formalin fixation. a Cross section of the fresh heart showing severe hypertrophy (20 mm LV free wall, 25 mm septum) with almost obliteration of the LV cavity. b Cross section of the formalin fixed heart confirming the findings in a) and clearly showing subendocardial and postero-septal scars. (PNG 54738 kb)

High Resolution (TIFF 17576 kb)

Supplemental Table 1 - updated table of Vanhaebost (DOCX 16.2 kb)

Supplemental Table 2 - children predicted normal ventricular wall thicknesses from birth to 19 years (Scholz et al, 1988). (DOCX 12 kb)

## Abbreviations

BSA: body surface area
CMR: cardiac magnetic resonance
CT: computed tomography
CTR: cardiothoracic ratio
DCM: dilated cardiomyopathy
HCM: hypertrophic cardiomyopathy
LV: left ventricle
LVH: left ventricular hypertrophy
LVNC: left ventricular noncompaction
LVOT: left ventricular outflow tract
PMCT: postmortem computed tomography
PMMR: postmortem magnetic resonance
RV: right ventricle
TEM: transmission electron microscopy

## References

[1] Cunningham KS, Spears DA, Care M (2019). Evaluation of cardiac hypertrophy in the setting of sudden cardiac death. Forensic Sci Res.

[2] Basso C, Aguilera B, Banner J, Cohle S, d'Amati G, de Gouveia RH, di Gioia C, Fabre A, Gallagher PJ, Leone O, Lucena J, Mitrofanova L, Molina P, Parsons S, Rizzo S, Sheppard MN, Mier MPS, Suvarna SK, Thiene G, van der Wal A, Vink A, Michaud K, Association for European Cardiovascular Pathology (2017). Guidelines for autopsy investigation of sudden cardiac death: 2017 update from the Association for European Cardiovascular Pathology. Virchows Arch.

[3] Elliott PM, Anastasakis A, Borger MA, Borggrefe M, Cecchi F, Charron P, Hagege AA, Lafont A, Limongelli G, Mahrholdt H, McKenna WJ, Mogensen J, Nihoyannopoulos P, Nistri S, Pieper PG, Pieske B, Rapezzi C, Rutten FH, Tillmanns C, Watkins H (2014). ESC Guidelines on diagnosis and management of hypertrophic cardiomyopathy: the Task Force for the Diagnosis and Management of Hypertrophic Cardiomyopathy of the European Society of Cardiology (ESC). Eur Heart J.

[4] Maron BJ, Towbin JA, Thiene G, Antzelevitch C, Corrado D, Arnett D, Moss AJ, Seidman CE, Young JB, American Heart Association, Council on Clinical Cardiology, Heart Failure and Transplantation Committee, Quality of Care and Outcomes Research and Functional Genomics and Translational Biology Interdisciplinary Working Groups, Council on Epidemiology and Prevention (2006). Contemporary definitions and classification of the cardiomyopathies: an American Heart Association Scientific Statement from the Council on Clinical Cardiology, Heart Failure and Transplantation Committee; Quality of Care and Outcomes Research and Functional Genomics and Translational Biology Interdisciplinary Working Groups; and Council on Epidemiology and Prevention. Circulation.

[5] Elliott P, Andersson B, Arbustini E, Bilinska Z, Cecchi F, Charron P, Dubourg O, Kühl U, Maisch B, McKenna WJ, Monserrat L, Pankuweit S, Rapezzi C, Seferovic P, Tavazzi L, Keren A (2008). Classification of the cardiomyopathies: a position statement from the European Society Of Cardiology Working Group on Myocardial and Pericardial Diseases. Eur Heart J.

[6] Vanhaebost J, Faouzi M, Mangin P, Michaud K (2014). New reference tables and user-friendly Internet application for predicted heart weights. Int J Legal Med.

[7] Ludwig J (1979) Current Methods of Autopsy Practice. C. W.B. Saunders, Editor

[8] Eckner FA, Brown BW, Overll E, Glagov S (1969). Alteration of the gross dimensions of the heart and its structures by formalin fixation. A quantitative study. Virchows Arch A Pathol Pathol Anat.

[9] Kitzman DW, Scholz DG, Hagen PT, Ilstrup DM, Edwards WD (1988). Age-related changes in normal human hearts during the first 10 decades of life. Part II (maturity): a quantitative anatomic study of 765 specimens from subjects 20 to 99 years old. Mayo Clin Proc.

[10] Scholz DG, Kitzman DW, Hagen PT, Ilstrup DM, Edwards WD (1988). Age-related changes in normal human hearts during the first 10 decades of life. Part I (growth): a quantitative anatomic study of 200 specimens from subjects from birth to 19 years old. [erratum appears in Mayo Clin Proc 1988 Jun;63(6):637]. Mayo Clin Proc.

[11] Maron BJ, Henry WL, Roberts WC, Epstein SE (1977). Comparison of echocardiographic and necropsy measurements of ventricular wall thicknesses in patients with and without disproportionate septal thickening. Circulation.

[12] Gray H (2016) Heart, in Gray’ Anatomy. 41st ed.: The anatomical basis of clinical practice, Elsevier, Editor. Churchill: London. p 998–1023

[13] Maleszewski JJ, Lai CK, Veinot JP (2016) Anatomic considerations and examination of cardiovascular specimens (excluding devices), in cardiovascular pathology Buja LM and Butany J, 4th edition, Elsevier, Editor. Amsterdam. p 1–56

[14] Jotterand M, Doenz F, Grabherr S, Faouzi M, Boone S, Mangin P, Michaud K (2016). The cardiothoracic ratio on post-mortem computer tomography. Int J Legal Med.

[15] Jotterand M, Faouzi M, Dédouit F, Michaud K (2019) New formula for cardiothoracic ratio for the diagnosis of cardiomegaly on post-mortem CT. Int J Legal Med 10.1007/s00414-019-02113-1 31346689

[16] Ampanozi G, Hatch GM, Flach PM, Thali MJ, Ruder TD (2015). Postmortem magnetic resonance imaging: reproducing typical autopsy heart measurements. Leg Med (Tokyo).

[17] Chatzaraki V, Thali MJ, Schweitzer W, Ampanozi G (2019). Left myocardial wall measurements on postmortem imaging compared to autopsy. Cardiovasc Pathol.

[18] Troxler R, Minoiu C, Vaucher P, Michaud K, Doenz F, Ducrot K, Grabherr S (2018). The role of angiography in the congruence of cardiovascular measurements between autopsy and postmortem imaging. Int J Legal Med.

[19] Jakobsen LS, Lundemose S, Banner J, Lynnerup N, Jacobsen C (2016). Forensic postmortem computed tomography: volumetric measurement of the heart and liver. Forensic Sci Med Pathol.

[20] Okuma H, Gonoi W, Ishida M, Shintani Y, Takazawa Y, Fukayama M, Ohtomo K (2013). Heart wall is thicker on postmortem computed tomography than on antemortem [corrected] computed tomography: the first longitudinal study. PLoS One.

[21] Gheorghe AG, Fuchs A, Jacobsen C, Kofoed KF, Møgelvang R, Lynnerup N, Banner J (2019). Cardiac left ventricular myocardial tissue density, evaluated by computed tomography and autopsy. BMC Med Imaging.

[22] Leone O, Veinot JP, Angelini A, Baandrup UT, Basso C, Berry G, Bruneval P, Burke M, Butany J, Calabrese F, d'Amati G, Edwards WD, Fallon JT, Fishbein MC, Gallagher PJ, Halushka MK, McManus B, Pucci A, Rodriguez ER, Saffitz JE, Sheppard MN, Steenbergen C, Stone JR, Tan C, Thiene G, van der Wal AC, Winters GL (2012). 2011 consensus statement on endomyocardial biopsy from the Association for European Cardiovascular Pathology and the Society for Cardiovascular Pathology. Cardiovasc Pathol.

[23] Mohammadi S, Hedjazi A, Sajjadian M, Ghoroubi N, Mohammadi M, Erfani S (2016). Study of the normal heart size in Northwest part of Iranian population: a cadaveric study. J Cardiovasc Thorac Res.

[24] Dadgar SK, Tyagi SP, Singh RP, Hameed S (1979). Factors influencing the normal heart weight--a study of 140 hearts. Jpn Circ J.

[25] de la Grandmaison GL, Clairand I, Durigon M (2001). Organ weight in 684 adult autopsies: new tables for a Caucasoid population. Forensic Sci Int.

[26] Gaitskell K, Perera R, Soilleux EJ (2011). Derivation of new reference tables for human heart weights in light of increasing body mass index. J Clin Pathol.

[27] Garby L, Lammert O, Kock KF, Thobo-Carlsen B (1993). Weights of brain, heart, liver, kidneys, and spleen in healthy and apparently healthy adult Danish subjects. Am J Hum Biol.

[28] Hanzlick R, Rydzewski D (1990). Heart weights of white men 20 to 39 years of age. An analysis of 218 autopsy cases. Am J Forensic Med Pathol.

[29] Hayes JAaL H (1966). Heart weight of Jamaicans: autopsy study of normal cases and cases of hypertension and chronic lung disease. Am Heart Assoc.

[30] Molina DK, DiMaio VJ (2012). Normal organ weights in men: part I-the heart. Am J Forensic Med Pathol.

[31] Ogiu N, Nakamura Y, Ijiri I, Hiraiwa K, Ogiu T (1997). A statistical analysis of the internal organ weights of normal Japanese people. Health Phys.

[32] Reiner L, Mazzoleni A, Rodriguez FL, Freudenthal RR (1959). The weight of the human heart. I Normal cases. AMA Arch Pathol.

[33] Smith HL (1928). The relation of the weight of the heart to the weight of the heart to age. Am Heart J.

[34] Yi-Suk Kim D-IK, Cho SY, Kim MH, Yang KM, Lee HY, Han S-H (2009). Statistical analysis for organ weights in Korean adult autopsies. Korean J Anat.

[35] Zeek PM (1942). Heart weight. I. The weight of the normal human heart. Arch Pathol.

[36] Seo JS, Lee SY, Won KJ, Kim DJ, Sohn DS, Yang KM, Cho SH, Park JD, Lee KH, Kim HD (2000). Relationship between normal heart size and body indices in Korean. J Korean Med Sci.

[37] Sheikhazadi A, Sadr SS, Ghadyani MH, Taheri SK, Manouchehri AA, Nazparvar B, Mehrpour O, Ghorbani M (2010). Study of the normal internal organ weights in Tehran’s population. J Forensic Legal Med.

[38] Skurdal AC, Nordrum IS (2016). A retrospective study of postmortem heart weight in an adult Norwegian population. Cardiovasc Pathol.

[39] Molina DK, DiMaio VJ (2015). Normal organ weights in women: part I-the heart. Am J Forensic Med Pathol.

[40] Sheppard MN (2011). Practical cardiovascular pathology.

[41] Weber KT (1989). Cardiac interstitium in health and disease: the fibrillar collagen network. J Am Coll Cardiol.

[42] Hill MA (2020) Embryology cardiac muscle histology. https://embryology.med.unsw.edu.au/embryology/index.php/Cardiac_Muscle_Histology. 09.01.2020. Available from: https://embryology.med.unsw.edu.au/embryology/index.php/Cardiac_Muscle_Histology.

[43] Varnava AM, Elliott PM, Sharma S, McKenna W, Davies MJ (2000). Hypertrophic cardiomyopathy: the interrelation of disarray, fibrosis, and small vessel disease. Heart.

[44] Davies MJ, McKenna WJ (1995). Hypertrophic cardiomyopathy--pathology and pathogenesis. Histopathology.

[45] Melacini P, Basso C, Angelini A, Calore C, Bobbo F, Tokajuk B, Bellini N, Smaniotto G, Zucchetto M, Iliceto S, Thiene G, Maron BJ (2010). Clinicopathological profiles of progressive heart failure in hypertrophic cardiomyopathy. Eur Heart J.

[46] Hughes SE (2004). The pathology of hypertrophic cardiomyopathy. Histopathology.

[47] Van Der Bel-Kahn J (1977). Muscle fiber disarray in common heart diseases. Am J Cardiol.

[48] McKenna WJ, Stewart JT, Nihoyannopoulos P, McGinty F, Davies MJ (1990). Hypertrophic cardiomyopathy without hypertrophy: two families with myocardial disarray in the absence of increased myocardial mass. Br Heart J.

[49] Basso C, Thiene G, Mackey-Bojack S, Frigo AC, Corrado D, Maron BJ (2009). Myocardial bridging, a frequent component of the hypertrophic cardiomyopathy phenotype, lacks systematic association with sudden cardiac death. Eur Heart J.

[50] Basso C, Thiene G, Corrado D, Buja G, Melacini P, Nava A (2000). Hypertrophic cardiomyopathy and sudden death in the young: pathologic evidence of myocardial ischemia. Hum Pathol.

[51] Davies MJ, McKenna WJ (1994). Dilated cardiomyopathy: an introduction to pathology and pathogenesis. Br Heart J.

[52] Waller BF (1988). Pathology of the cardiomyopathies. J Am Soc Echocardiogr.

[53] Rose AG, Beck W (1985). Dilated (congestive) cardiomyopathy: a syndrome of severe cardiac dysfunction with remarkably few morphological features of myocardial damage. Histopathology.

[54] Kuroda, K (2015) Hypertensive cardiomyopathy: A clinical approach and literature review. World Journal of Hypertension 5(2):41–52. 10.5494/wjh.v5.i2.41

[55] Zia MI, Valenti V, Cherston C, Criscito M, Uretsky S, Wolff S (2012). Relation of mitral valve prolapse to basal left ventricular hypertrophy as determined by cardiac magnetic resonance imaging. Am J Cardiol.

[56] Basso C, Perazzolo Marra M, Rizzo S, de Lazzari M, Giorgi B, Cipriani A, Frigo AC, Rigato I, Migliore F, Pilichou K, Bertaglia E, Cacciavillani L, Bauce B, Corrado D, Thiene G, Iliceto S (2015). Arrhythmic mitral valve prolapse and sudden cardiac death. Circulation.

[57] Basso C, Thiene G, Corrado D, Angelini A, Nava A, Valente M (1996). Arrhythmogenic right ventricular cardiomyopathy. Dysplasia, dystrophy, or myocarditis?. Circulation.

[58] Gerçek M, Gerçek M, Kant S, Simsekyilmaz S, Kassner A, Milting H, Liehn EA, Leube RE, Krusche CA (2017). Cardiomyocyte hypertrophy in arrhythmogenic cardiomyopathy. Am J Pathol.

[59] Miles C, Finocchiaro G, Papadakis M, Gray B, Westaby J, Ensam B, Basu J, Parry-Williams G, Papatheodorou E, Paterson C, Malhotra A, Robertus JL, Ware JS, Cook SA, Asimaki A, Witney A, Ster IC, Tome M, Sharma S, Behr ER, Sheppard MN (2019). Sudden death and left ventricular involvement in arrhythmogenic cardiomyopathy. Circulation.

[60] Sun L, Yu M, Zhou T, Zhang S, He G, Wang G, Gang X (2019). Current advances in the study of diabetic cardiomyopathy: from clinicopathological features to molecular therapeutics (Review). Mol Med Rep.

[61] Avelar E, Cloward TV, Walker JM, Farney RJ, Strong M, Pendleton RC, Segerson N, Adams TD, Gress RE, Hunt SC, Litwin SE (2007). Left ventricular hypertrophy in severe obesity: interactions among blood pressure, nocturnal hypoxemia, and body mass. Hypertension.

[62] Angelini A, di Gioia C, Doran H, Fedrigo M, de Gouveia RH, Ho SY, Leone O, Sheppard MN, Thiene G, Dimopoulos K, Mulder B, Padalino M, van der Wal AC, Association for European Cardiovascular Pathology (AECVP) (2020). Autopsy in adults with congenital heart disease (ACHD). Virchows Arch.

[63] Lucena J, Blanco M, Jurado C, Rico A, Salguero M, Vazquez R, Thiene G, Basso C (2010). Cocaine-related sudden death: a prospective investigation in south-west Spain. Eur Heart J.

[64] Havakuk O, Rezkalla SH, Kloner RA (2017). The cardiovascular effects of cocaine. J Am Coll Cardiol.

[65] Karch SB, DO (2016) Synthetic stimulants. In: Karchs’s pathology of drugs of abuse, 5th ed, B.R. CRC Press, Editor. FL. p 273–364

[66] Baggish AL, Weiner RB, Kanayama G, Hudson JI, Lu MT, Hoffmann U, Pope HG (2017). Cardiovascular toxicity of illicit anabolic-androgenic steroid use. Circulation.

[67] Maron BJ, Pelliccia A (2006). The heart of trained athletes: cardiac remodeling and the risks of sports, including sudden death. Circulation.

[68] Sharma S, Drezner JA, Baggish A, Papadakis M, Wilson MG, Prutkin JM, la Gerche A, Ackerman MJ, Borjesson M, Salerno JC, Asif IM, Owens DS, Chung EH, Emery MS, Froelicher VF, Heidbuchel H, Adamuz C, Asplund CA, Cohen G, Harmon KG, Marek JC, Molossi S, Niebauer J, Pelto HF, Perez MV, Riding NR, Saarel T, Schmied CM, Shipon DM, Stein R, Vetter VL, Pelliccia A, Corrado D (2018). International recommendations for electrocardiographic interpretation in athletes. Eur Heart J.

[69] Spence JD, Rayner BL (2018). Hypertension in blacks: individualized therapy based on renin/aldosterone phenotyping. Hypertension.

[70] Basavarajaiah S, Boraita A, Whyte G, Wilson M, Carby L, Shah A, Sharma S (2008). Ethnic differences in left ventricular remodeling in highly-trained athletes relevance to differentiating physiologic left ventricular hypertrophy from hypertrophic cardiomyopathy. J Am Coll Cardiol.

[71] Goor D, Lillehei CW, Edwards JE (1969). The “sigmoid septum”. Variation in the contour of the left ventricular outlet. Am J Roentgenol Radium Therapy, Nucl Med.

[72] Kayvanpour E, Sedaghat-Hamedani F, Gi WT, Tugrul OF, Amr A, Haas J, Zhu F, Ehlermann P, Uhlmann L, Katus HA, Meder B (2019). Clinical and genetic insights into non-compaction: a meta-analysis and systematic review on 7598 individuals. Clin Res Cardiol.

[73] Towbin JA, Lorts A, Jefferies JL (2015). Left ventricular non-compaction cardiomyopathy. Lancet.

[74] Arbustini E, Favalli V, Narula N, Serio A, Grasso M (2016). Left ventricular noncompaction: a distinct genetic cardiomyopathy?. J Am Coll Cardiol.

[75] Finocchiaro G, Dhutia H, Gray B, Ensam B, Papatheodorou S, Miles C, Malhotra A, Fanton Z, Bulleros P, Homfray T, Witney AA, Bunce N, Anderson LJ, Ware JS, Sharma R, Tome M, Behr ER, Sheppard MN, Papadakis M, Sharma S (2020). Diagnostic yield of hypertrophic cardiomyopathy in first-degree relatives of decedents with idiopathic left ventricular hypertrophy. Europace.

[76] Finocchiaro G, Papadakis M, Robertus JL, Dhutia H, Steriotis AK, Tome M, Mellor G, Merghani A, Malhotra A, Behr E, Sharma S, Sheppard MN (2016). Etiology of sudden death in sports: insights from a United Kingdom regional registry. J Am Coll Cardiol.

[77] Hiramitsu S, Morimoto SI, Kato S, Uemura A, Kubo N, Kimura K, Sugiura A, Itoh T, Hishida H (2001). Transient ventricular wall thickening in acute myocarditis: a serial echocardiographic and histopathologic study. Jpn Circ J.

[78] Turschner O, D'hooge J, Dommke C, Claus P, Verbeken E, de Scheerder I, Bijnens B, Sutherland GR (2004). The sequential changes in myocardial thickness and thickening which occur during acute transmural infarction, infarct reperfusion and the resultant expression of reperfusion injury. Eur Heart J.

[79] Sabatasso S, Banz Y, Ringger R, Visonà S, Schyma C, Bolliger S, Michaud K (2020). Second opinion system for sudden cardiac death cases in forensic practice. Int J Legal Med.

[80] Fellmann F, van El CG, Charron P, Michaud K, Howard HC, Boers SN, Clarke AJ, Duguet A-M, Forzano F, Kauferstein S, Kayserili H, Lucassen A, Mendes Á, Patch C, Radojkovic D, Rial-Sebbag E, Sheppard MN, Tassé A-M, Temel SG, Sajantila A, Basso C, Wilde AAM, Cornel MC (2019). European recommendations integrating genetic testing into multidisciplinary management of sudden cardiac death. Eur J Hum Genet.

