# Supplementary figures and images for: Cardiac hypertrophy at autopsy
Source: Virchows Arch. 2021 Mar 19;479(1):79–94. doi: 10.1007/s00428-021-03038-0 (PMC8298245; doi:10.1007/s00428-021-03038-0)

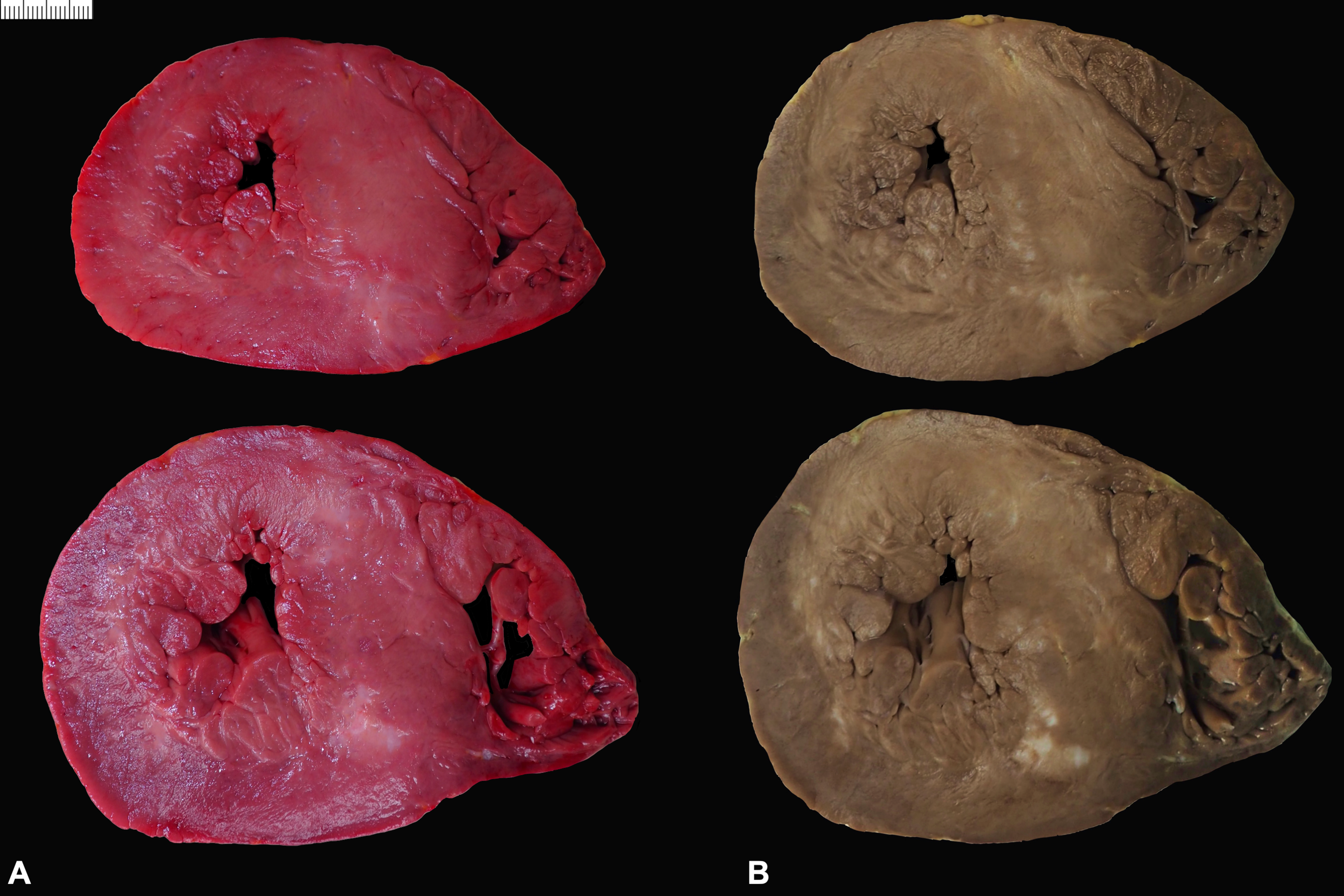

Supplement: Supplementary file 1 — Hypertrophic cardiomyopathy, gross examination of the heart in the autopsy room and after formalin fixation. a Cross section of the fresh heart showing severe hypertrophy (20 mm LV free wall, 25 mm septum) with almost obliteration of the LV cavity. b Cross section of the formalin fixed heart confirming the findings in a) and clearly showing subendocardial and postero-septal scars. (PNG 54738 kb) [file 428_2021_3038_Fig13_ESM.png]

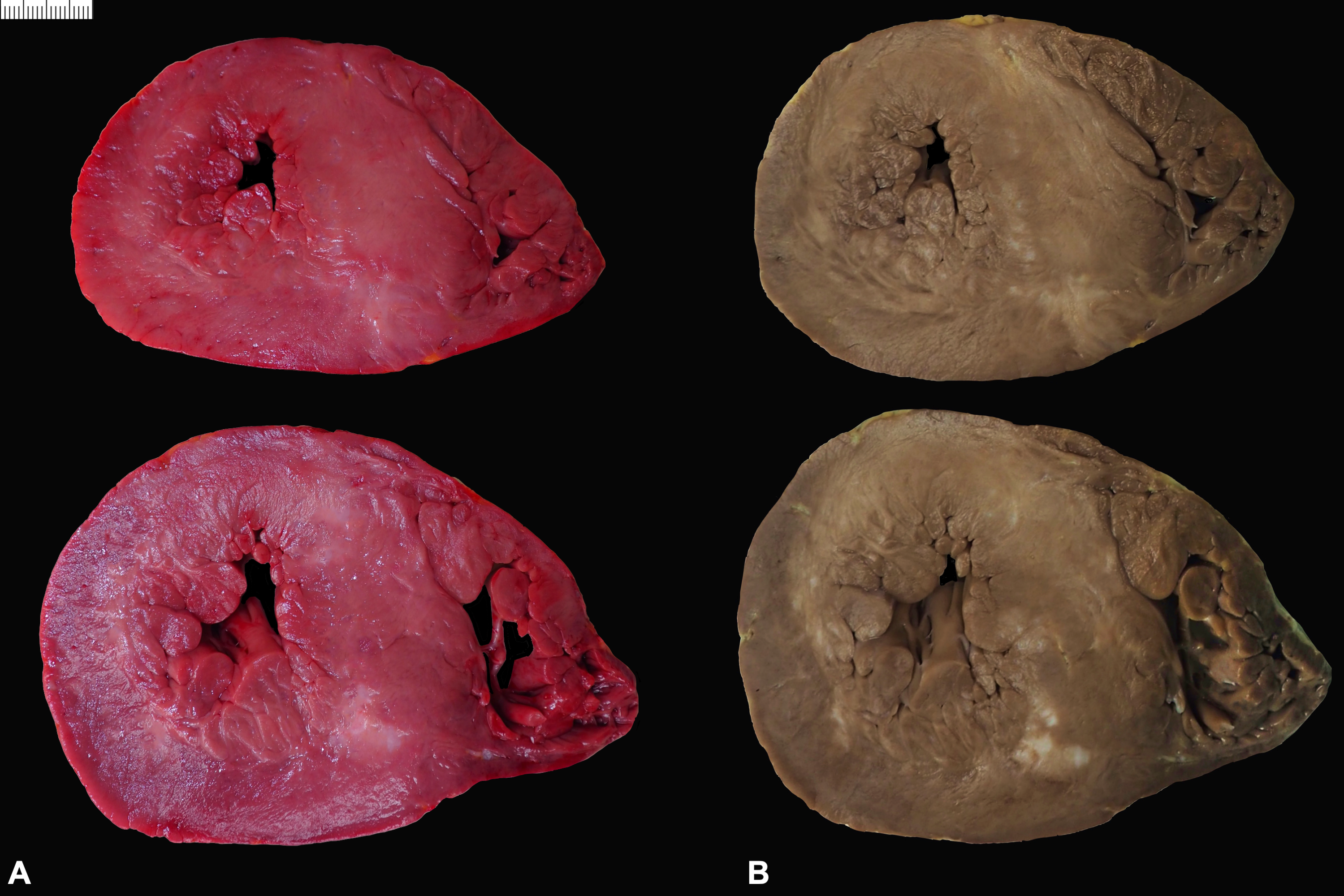

Supplement: Supplementary file 2 — High Resolution (TIFF 17576 kb) [file 428_2021_3038_MOESM1_ESM.tiff]
