# Supplementary material for: Cardiac hypertrophy at autopsy
Source: Virchows Arch. 2021 Mar 19;479(1):79–94. doi: 10.1007/s00428-021-03038-0 (PMC8298245; doi:10.1007/s00428-021-03038-0)
Supplement: Supplementary file 3 — - updated table of Vanhaebost (DOCX 16.2 kb) [file 428_2021_3038_MOESM2_ESM.docx]

| **Study** | **Year of publication** | **Period of the study** | **Country/population** | **Number of cases** | **Age range (mean), yr** | | **Heart weight mean (SD), g** | | **Heart weight range, g** | | **Predictor*** |
| --- | --- | --- | --- | --- | --- | --- | --- | --- | --- | --- | --- |
|  |  |  |  |  | **Men** | **Women** | **Men** | **Women** | **MEN** | **WOMEN** |  |
| Horace - Arnold | 1899 | 1894 - 1898 | USA, hospital cases | 216 (134 M, 82 W) |  |  | 290 | 253 | 250 - 325 | 225 - 300 |  |
| Smith | 1928 |  | USA, hospital cases | 854 (534 M, 320 W) | 18-80 | 18-80 | 294 | 250 | 137 - 400 | 110 - 375 | BW |
| Zeek | 1942 | 1924 - 1940 | USA, hospital cases | 926 (523 M, 403 F) | 21 - 69 | 21 - 49 | 312 (52) | 252.9 (46.4) | 200 - 424 | 150 - 374 | BL |
| Reiner | 1959 | 1953 - 1955 | USA | 45 (26 M, 19 w) | 34 - 92 | 21 - 68 | 328 | 244 | 256 - 390 | 198 - 279 |  |
| Hayes | 1966 | j1962 - 1962 | Jamaica | 58 (30 m, 28 W) | 43.5 (14) | 43.5 (16.9) | 294.5 (48.5) | 258.5 (49.5) |  |  |  |
| Dadgar | 1979 | unknown | India | 138 (116 M, 24 W) | 0.003 to 78 |  | 236.1 (56.81) | 206.6 (78.61) | 60 - 375 | 25 - 375 | BL |
| Kitzman | 1988 | 1960 - 1982 | USA, hospital cases | 765 (373 M, 392 W) | 20 - 99 | |  |  | 164 - 557 | | BW > BSA > BH |
| Hanzlick | 1990 |  | men | 201 | 20 - 39 |  | 364 (62) |  |  |  | age and BW |
| Garby | 1993 | 1972 - 1990 | Danish | 964 (630 M, 334 W) | 45 (17) | 41 (18) | 423 (87) | 320 (67) |  |  |  |
| Ogiu | 1997 | 1985 - 1989 | Japan | 4667 (3023 M, 1644 W) | 0 - 95 | 0 - 93 | 292 - 321 (38 - 51) |  |  |  |  |
| Seok Seo | 2000 | 1994 - 1998 | Korea | 422 (215 m, 207 W) | 1 to 76 | 0 - 77 | 305 | 265 | 280 - 340 | 230 - 280 | BSA > BW > others |
| De la Grandmaison | 2001 | 1987 - 1991 | France, forensic cases | 684 (355 M, 329 W) | 42 (17) | 49 (20) | 365 (71) | 312 (78) | 90 - 630 | 174 - 590 | age-BW-BMI>BH |
| da Cunha | 2002 | 1986 - 1998 | Brasil | 21 | 44.7 (21.8) | | 329.1 (50.4) | |  |  | BW>BMI |
| Yi-Suk Kim | 2009 | 2003 - 2005 | Korea | 526 (369 M, 157 W) | 43.4 (12.54) | 44.6 (14.75) | 346.81 (57.90) | 298.79 (62.59) |  |  |  |
| Sheikhazadi | 2010 | 2007 - 2008 | Tehran | 1222 (914 M, 308 W) | 43.4 (17.8) | 45.2 (22.2) | 359.9 (76.6) | 319.2 (86.2) | 209 - 607 | 199 - 540 | BMI > BH |
| Gaitskell | 2010 | 2003 - 2006 | United Kingdom, hospital cases | 384 (204 M, 180 W) | 14 - 98 | | 380 | 329 | 192 - 672 | 197 - 765 | BSA > BW > others |
| Molina - DiMaio | 2012 | 2005 - 2011 | USA, forensic cases | 232 men | 18 - 35 | 0 | 331 (56.7) |  | 188 - 575 |  |  |
| Vanhebost | 2013 | 2007 - 2011 | Switzerland, forensic cases | 288 (170 M, 118 W) | 37.08 (13.41) | 42.2 (15.7) | 357.1 (53) | 289.8 (63.22) | 260 - 550 | 160 - 455 | BSA > BW > others |
| Wingren | 2015 | 1997-2013 | Sweden, forensic cases | 27 645 (20144 M, 7501 W) | adults | adults | 386.2 (101–994) | 386.2 (101–994) | 103-985 | | BH, BSA and BW  in a normal to overweighed  population |

**Suppl Table 1**. Studies of a normal heart weight (M men; W, women; BL body length; BH, body height; BW, body weight; BSA, body surface area, BMI, body mass index
