# Supplementary material for: Cardiac hypertrophy at autopsy
Source: Virchows Arch. 2021 Mar 19;479(1):79–94. doi: 10.1007/s00428-021-03038-0 (PMC8298245; doi:10.1007/s00428-021-03038-0)
Supplement: Supplementary file 4 — - children predicted normal ventricular wall thicknesses from birth to 19 years (Scholz et al, 1988). (DOCX 12 kb) [file 428_2021_3038_MOESM3_ESM.docx]

**Supplemental Table 2** – children predicted normal ventricular wall thicknesses from birth to 19 years Scholz et al 1988

| Age | Gender | Right ventricle | Left ventricle | Ventricular septum |
| --- | --- | --- | --- | --- |
| first year | Female  Male | 0.20 (0.34)  0.16 (0.31) | 0.51(0.78)  0.41 (0.71) | 0.62 (0.93)  0.50 (0.83) |
| 10 years | Female  Male | 0.30 (0.45)  0.33 (0.50) | 0.95 (1.21)  1.03 (1.33) | 0.99 (1.31)  1.13 (1.47) |
| 19 years | Female  Male | 0.32 (0.47)  0.38 (0.55) | 1.04 (1.31)  1.18 (1.47) | 1.08 (1.40)  1.27 (1.61) |

Values are expressed as mean (and upper 95%) in mm
